# Assessment of Purity, Stability, and Pharmacokinetics of NGP-1, a Novel Prodrug of GS441254 with Potential Anti-SARS-CoV-2 Activity, Using Liquid Chromatography

**DOI:** 10.3390/molecules28155634

**Published:** 2023-07-25

**Authors:** Chen Sun, Bo Liu, Fengzhi Zhou, Qianqian Zheng, Chunmei Dai, Wei Wei, Guochao Liao, Yuqi Sun

**Affiliations:** 1 School of Pharmacy, Jinzhou Medical University, Jinzhou 121001, China; 2 International Institute for Translational Chinese Medicine, Guangzhou University of Chinese Medicine, Guangzhou 510006, China

**Keywords:** anti-SARS-CoV-2, prodrug of GS441524, purity, stability and pharmacokinetics, oral bioavailability, LC-MS/MS

## Abstract

SARS-CoV-2 is a highly contagious and pathogenic virus that first appeared in late December 2019 and caused a global pandemic in a short period. The virus is a single-stranded RNA virus belonging to the Coronaviridae family. Numerous treatments have been developed and tested in response to the pandemic, particularly antiviral drugs. Among them, GS441524 (GS441), a nucleoside antiviral drug, has demonstrated promising results in inhibiting SARS-CoV-2. Nevertheless, the limited oral bioavailability of GS441 restricts its application to patients with the virus. In this study, a novel prodrug of GS441 (NGP-1) with an isobutyl ester and cyclic carbonate structure was designed and synthesized. Its purity and the stability in different artificial digestive juices of NGP-1 was determined with HPLC-DAD methods. The pharmacokinetics of NGP-1 and GS441 were studied in rats via gavage administration. A new LC-MS/MS method was developed to quantitatively analyze GS441 in plasma samples. The results showed that the k_a_, C_max_, and MRT of converted GS441 from NGP-1 were 5.9, 3, and 2.5 times greater than those of GS441 alone. The F_rel_ of NGP-1 was approximately four-fold that of GS441, with an AUC_0–∞_ of 9716.3 h·ng mL^−1^. As a prodrug of GS441, NGP-1 increased its lipophilicity, absorption, and bioavailability, indicating that it holds promise in improving the clinical efficacy of anti-SARS-CoV-2 medications.

## 1. Introduction

Severe acute respiratory syndrome coronavirus 2 (SARS-CoV-2) emerged in 2019 and caused a global pandemic [1]. The virus’ spread was unprecedentedly fast, diffusing to more than 180 countries within 3 months [2]. The World Health Organization (WHO) has, to date, documented nearly 767 million cases and more than 6.94 million fatalities worldwide. In May 2023, the WHO announced that the epidemic no longer met the criteria to be considered a public health emergency of international concern. Nevertheless, specialists anticipate that the SARS-CoV-2 pandemic will persist, as occasional cases of SARS-CoV-2 are still recorded, requiring effective therapy. The coronaviruses are spherical, enveloped, and single-stranded RNA viruses susceptible to easy and random genetic variations [3]. Many mutations have been reported during the last 3 years. The five mutant strains most frequently reported are Alpha, Beta, Gamma, Delta, and Omicron [4]. The evolution of the virus and the global emergence of variant strains have affected its transmissibility, virulence, and the severity of its clinical manifestations [5]. The huge global implications of the pandemic indicate the necessity to continue vaccine research and development, as safe and effective drugs are still needed to control and treat SARS-CoV-2 infections.

**Figure 1:**
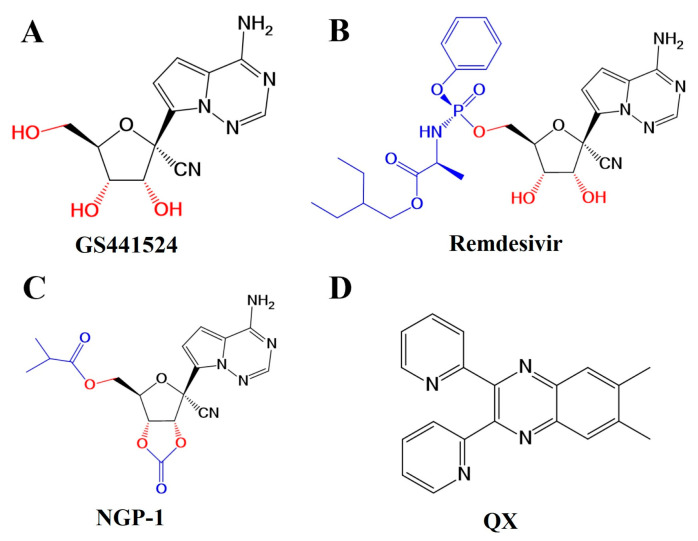
Molecular structures of GS441, different prodrugs, and internal standard. (**A**) GS441, (**B**) RDV, (**C**) NGP-1, (**D**) QX.

A broad-spectrum anti-coronaviral drug acting as viral RNA could play a significant role in fighting the pandemic. It should be effective against different variants of SARS-CoV-2. Nucleotide analogs show considerable promise as antiviral drugs. Many of them have been approved and become candidates for the treatment of SARS-CoV-2 [6]. GS441524 (GS441, Figure 1A) is an adenosine analog investigated for its use in treating SARS-CoV-2 infections. It is phosphorylated three times to form the active nucleoside triphosphate through the action of cellular kinases, and it is incorporated into the genomes of virions, terminating its replication and inhibiting viral RNA synthesis [7,8]. The activity of GS441 is supported by findings that it markedly inhibits SARS-CoV-2 in cell lines and possesses anti-SARS-CoV-2 activity in mouse models [9]. However, its molecular structure exhibits significant polarity and hydrogen-bonding capabilities. These properties limit the biomembrane transport and gastrointestinal absorption, rendering it unsuitable for oral administration [10]. As a prodrug of an adenine nucleotide analog, remdesivir (GS-5734, RDV, Figure 1B) can be metabolized into GS441 and triphosphorylated in vivo. In vitro studies have revealed that GS441 exhibits a higher EC_50_ than RDV against several viruses, indicating that RDV is more potent [11]. Upon the intravenous injection of RDV in rhesus monkeys, it was rapidly distributed into peripheral blood mononuclear cells (PBMCs) and converted into active nucleoside triphosphate [12]. The converted GS441 demonstrated a longer presence and sustained activity. It was the first FDA-approved antiviral treatment drug for SARS-CoV-2 [13]. Nevertheless, a significant drawback of RDV is that it has strong liver metabolism and even leads to almost complete first-pass clearance. It is not highly bioavailable following oral administration. It requires intravenous administration, limiting its clinical use [14]. Oral administration is a more expedient, secure, and efficacious mode of delivery compared with injection. The nucleosides must traverse the cell membrane, where they are progressively phosphorylated into active metabolites by intracellular enzymes [15]. The nucleoside analogs often exhibit insufficient cell phosphorylation and low bioactivity due to weak membrane permeability [16]. The hydrophilic properties limit the cell membrane penetration ability of the absorption sites. Therefore, the development of a highly active, orally bioavailable analog of GS441 is urgently needed.

To this end, NGP-1 (Figure 1C), a novel GS441 prodrug, has been designed, anticipated to provide an oral treatment option with potential anti-SARS-CoV-2 activity. The GS441 molecule underwent conversion into a cyclic carbonic ester structure. Additionally, the hydroxyl side chain of GS441 was esterified with an isobutyl group. These modifications were expected to enhance the lipophilicity of the compound and increase its ability to penetrate the cell membrane, ultimately leading to improved oral bioavailability. In this study, various HPLC methods were developed and used to evaluate NGP-1. First, the purity of NGP-1 was assessed using HPLC-DAD analysis. Subsequently, the stability of NGP-1 was tested using HPLC-DAD under different pH conditions with artificial digestive solutions. Furthermore, an LC-MS/MS method was established to determine the concentration of GS441 in blood samples, enabling the evaluation of the pharmacokinetic characteristics of NGP-1. Overall, the use of HPLC methods in this study facilitated the evaluation of NGP-1 in terms of purity, stability, and pharmacokinetic properties. The findings of this study provide insights for further studies exploring the efficacy of the modified GS441 molecule as a potential therapeutic agent.

## 2. Results and Discussion

### 2.1. Synthesis of GS441 Cyclic Carbonate Prodrug

GS441 possesses the ability to interfere with the activity of viral RNA-dependent RNA polymerases, thus inhibiting viral RNA synthesis [17]. However, its oral absorption is limited due to its poor liposolubility. A prodrug strategy was implemented to develop a new compound to increase its lipid solubility, enhance its membrane permeability, and improve its bioavailability. Ester prodrugs are predominantly used to enhance the lipophilicity of polar drugs by masking charged functional groups such as carboxylic acid and hydroxyl groups [18]. Among these, cyclic carbonate prodrugs are inherently unstable in human plasma and do not require esterases for cellular metabolism, allowing the release of active ingredients [10]. Therefore, we formulated and synthesized the NGP-1 prodrug with both isobutyl ester and cyclic carbonate structures based on the molecular structure of GS441.

TBSCl was chosen as a protective agent for one hydroxyl group, while a cyclic carbonate structure was formed in a further step during the synthesis of NGP-1 [19]. CDI was selected due to its superior reactivity, easy handling, cost-effectiveness, and toxicity-reducing properties compared with triphosgene or ethyl chloroformate [20,21]. Additionally, DMAP, an exceptionally efficient catalyst, was used during the low-temperature dehydration process to synthesize the side chain isobutyl ester.

**Figure 2:**
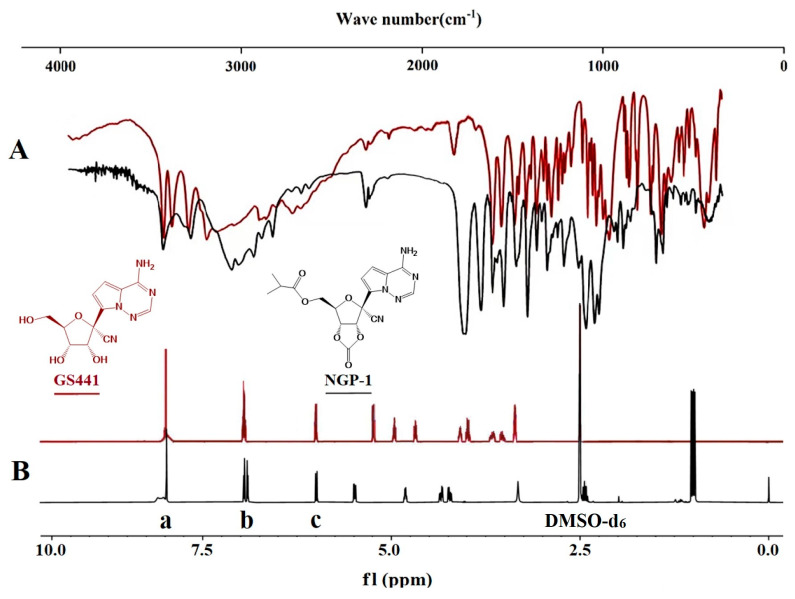
Structural characteristics of GS441 and NGP-1 using FTIR and ^1^H-NMR spectra. (**A**) FTIR of GS441 and NGP-1, (**B**) ^1^H-NMR of GS441 and NGP-1.

As depicted in Figure 2A, the spectral signal of GS441 at 1335 cm^−1^ originated from the in-plane bending vibration of -OH. Moreover, the observed peaks at 1136 cm^−1^ and 1150 cm^−1^ corresponded to absorption peaks resulting from stretching vibrations of C-N. The strong absorption peaks observed at 1087 cm^−1^ and 1100 cm^−1^ were attributed to stretching vibrations of the fatty ether C-O-C bonds. In the case of NGP-1, the specific spectral bands were identified at 1728 cm^−1^ and 1150 cm^−1^, representing stretching vibration signals of C=O and C-O (C-O-C), respectively. These signals indicated the presence of ester bonds. The dual frequency peak of C=O was observed at 3430 cm^−1^, whereas the strong absorption peak at 1813 cm^−1^ represented the stretching vibration of C=O in cyclic anhydride. In contrast, no absorption is discernible in Figure 2A, suggesting the existence of a cyclic carbonate structure.

Further structural analysis of the drugs was carried out in DMSO-d_6_ through NMR spectroscopy. The ^1^H-NMR spectrum, presented in Figure 2B, revealed that the signal at δ = 3.94 ppm (dd, *J* = 5.9, 4.3 Hz, 2H) originated from the hydrogen of -OH on the side chain in GS441. Further, the peak at δ = 1.12 ppm (dd, *J* = 7.0, 1.6 Hz, 6H) was generated from the hydrogen of -CH_3_ on the side chain in NGP-1. The signals detected at δ = 2.40 ppm (q, *J* = 14.0, 7.0 Hz, 1H) and δ = 4.32 ppm (q, *J* = 4.3 Hz, 1H) corresponded to the hydrogen of -CH_2_-, indicating that an isobutyl ester side chain had been formed. The peaks detected at δ = 4.27 ppm (q, *J* = 5.9 Hz, 1H) and δ = 4.67 ppm (t, *J* = 4.2 Hz, 1H) were primarily attributed to the hydrogen of ethylene carbonate, providing evidence that the cyclic carbonate structure had been successfully synthesized. The peaks labeled as a (s, 1H), b (dd, *J* = 4.7 Hz, 1H), and c (d, *J* = 4.7 Hz, 1H) corresponded to the hydrogen of 1,2,4-triazine, -NH_2_, and -CH from the pyrrole structure, respectively.

**Figure 3:**
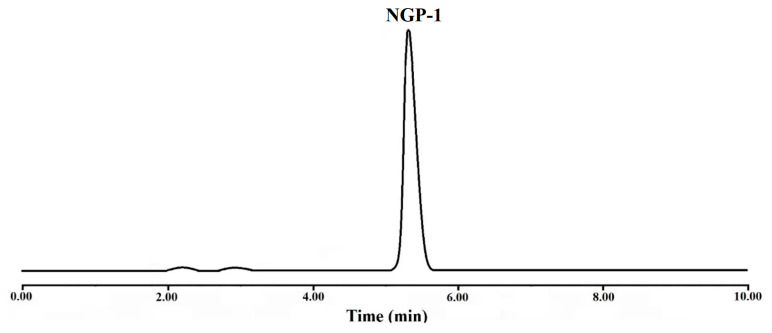
HPLC-DAD chromatogram of NGP-1 at 240 nm used for purity evaluation.

The identification of NGP-1 was confirmed by IR and NMR analysis. Additionally, the HPLC peak area normalization method was employed to study the purity of the newly generated compound. As shown in Figure 3, the peak at approximately 5.38 min exhibited the most significant peak area. Consequently, the new GS441 prodrug (NGP-1), featuring a cyclic carbonate structure and an isobutyl ester side chain, was successfully synthesized, with a yield of 25.6% and a purity level exceeding 98.0%.

### 2.2. Investigation of the Stability of NGP-1 in Artificial Digestive Juices

Esterification is the predominant reaction for the generation of prodrugs, owing to the facile hydrolysis of esters by ubiquitous esterases present in the various matrixes [18,22]. However, esters, particularly cyclic lactones, may cause instability in acidic, basic, or esterase environments. In oral administration, the drugs pass through absorption sites, such as the stomach and small intestine, encountering varying pH conditions. In this study, HPLC-DAD was employed to assess the stability of NGP-1 in different artificial digestive juices with different pH levels [23,24].

**Figure 4:**
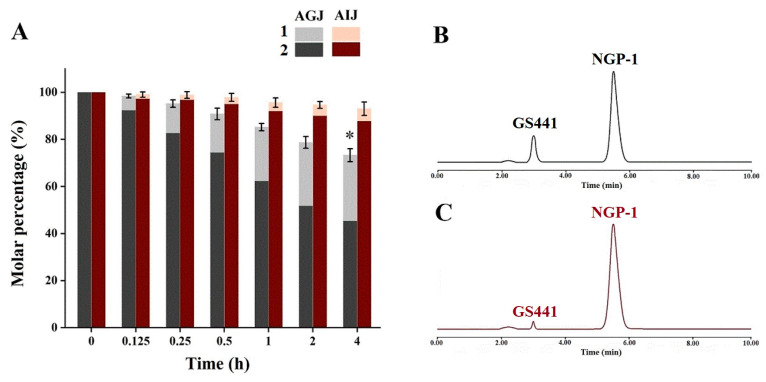
Stability of NGP-1 in artificial gastric juice (AGJ) and artificial intestinal juice (AIJ). (**A**) Molar percentage of NGP-1 and converted GS441 in digestive juices; (**B**) HPLC chromatogram of NGP-1 in AGJ (1 h); (**C**) HPLC chromatogram of NGP-1 in AIJ (1 h). (*) compared with 0 h group, * *p* < 0.05.

As shown in Figure 4, NGP-1 exhibited relatively stable behavior when subjected to artificial intestinal juice at pH 6.8. However, the degradation rate of NGP-1 was observed to accelerate significantly when exposed to artificial gastric juice. The conversion rate of NGP-1 increased obviously with time, with an increase in the acidity level. Hence, the degradation rate of NGP-1 was much slower in neutral conditions than in acidic conditions. 

The artificial gastric juice exhibited increased aggression with a lower pH, resulting in structural changes in NGP-1. Moreover, a significant difference was observed in the total amount of converted GS441 and NGP-1 between 4 and 0 h in artificial gastric juice. The total amount of converted GS441 and NGP-1 decreased gradually in the artificial gastric juice, implying the conversion of other components besides GS441 under acidic conditions. The cleavage of ester bonds usually occurs through hydrolysis or oxidation in vivo. Ester bonds are highly unstable due to the widespread distribution of esterases throughout the body [10]. These results suggest that some NGP-1 could be directly converted into GS441 in the stomach, whereas the remaining NGP-1 could be converted into GS441 on entering the blood after oral administration. 

### 2.3. Pharmacokinetic Study

**Figure 5:**
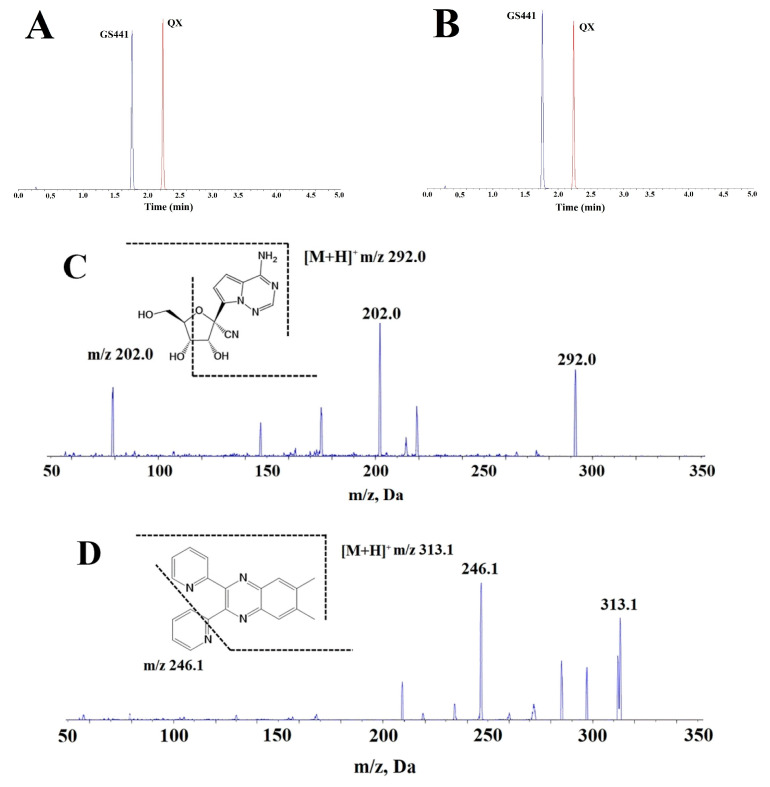
Typical MRM chromatograms of pharmacokinetic samples using information from product ion mass spectra. (**A**) reference chromatogram (GS441: 200 ng/mL, QX: 20 ng/mL); (**B**) sample chromatogram (0.5 h, QX: 20 ng/mL); (**C**) product ion mass spectra of GS441; (**D**) product ion mass of QX.

In this study, the quantity of GS441 was measured using the LC-MS/MS method in the collected plasma samples. Additionally, 6,7-dimethyl-2,3-bis(2-pyridyl) quinoxaline (QX, Figure 1D) was selected as an internal standard (IS) due to its good stability and strong MS detection response [12]. The multiple reaction monitoring (MRM) transitions and the typical chromatograms of GS441 and QX are depicted in Figure 5A–D. An LC-MS/MS method was developed and validated for the determination of GS441 in the plasma samples from experimental rats (Supplementary Materials). The analysis showed high precision and accuracy. The peaks displayed high symmetry without any signal interference. The calibration equation for GS441 was y = 0.0025x − 0.0017 (r = 0.9993, 1–800 ng/mL), where x and y represented the concentration of GS441 in rat plasma and the peak area ratio of analyte to QX, respectively.

Notably, the cellular uptake of GS441 could depend on membrane-binding transporters due to its hydrophilicity, which limits its absorption from the gastrointestinal tract [25]. Prodrugs may overcome various barriers to drug formulation and delivery, such as insufficient oral absorption [26]. Esterification is a widely researched reaction for the synthesis of prodrugs containing active compounds and drugs with carboxylic acids and free hydroxyl groups. This reaction generally alters the pharmacokinetic properties [27]. It is undeniable that carbon esters have a tendency to produce aldehyde and ketone metabolites in vivo, which may potentially pose toxicity to the body, such as the kidneys and the excretory, neurological, and immune systems [28]. This aspect will undoubtedly be an essential component of future studies.

**Figure 6:**
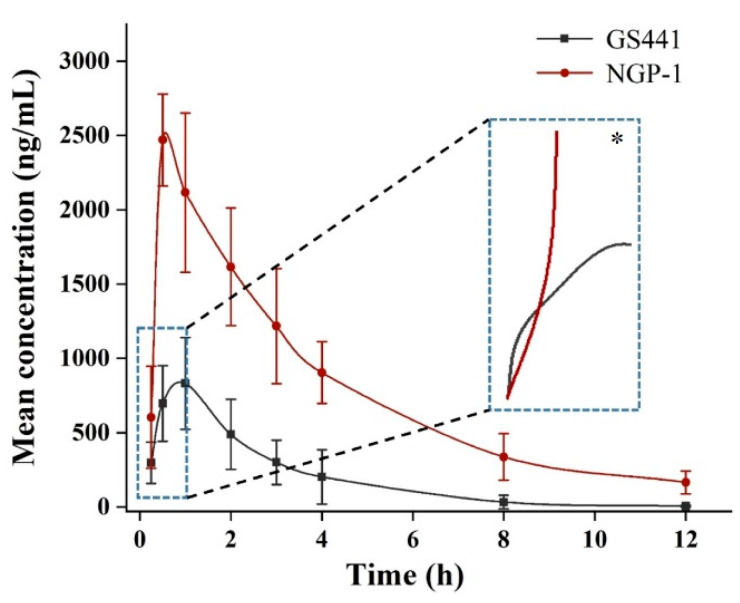
Mean plasma concentration–time curves of converted GS441 from NGP-1 and GS441 after intragastric administration of NGP-1 and GS441 in rats, respectively. (n = 6). * Predictive GS441 concentration in the initial absorption phase.

Blood samples were collected and the plasma concentration of GS441 was determined using the LC-MS/MS technique following the intragastric administration of GS441 and NGP-1 in rats, respectively. Figure 6 illustrates the concentration–time curve, and the pharmacokinetic parameters were set as presented in Table 1. The curve of GS441 presented a relatively smooth pattern after the intragastric administration of equimolar amounts of NGP-1 and GS441. The peak concentration of GS441 was lower than that of NGP-1, and the elimination was more complete after 12 h. Compared with GS441, the converted GS441 rapidly reached a higher concentration after the oral administration of NGP-1. NGP-1 could be partially converted into GS441 due to its instability under the gastric acid environment. Therefore, during the initial stage of absorption, the concentration of GS441 consisted of both the absorption of converted GS441 in the stomach and the absorption of NGP-1 converted into GS441 in the blood. The oral administration of NGP-1 consistently maintained the high required concentration of GS441 during the elimination phase.

Table 1 displays the pharmacokinetic parameters of GS441 and the converted GS441 from NGP-1 in vivo. The absorption rate constant (k_a_) of the converted GS441 from NGP-1 was found to be 5.9 times greater than that of GS441 alone, implying the faster absorption of NGP-1 compared with GS441, when administered orally. We found that the lipophilic NGP-1 was rapidly absorbed into the systemic circulation and swiftly converted into GS441. The converted GS441 from NGP-1 reached a C_max_ of 2470.3 ng/mL, which was approximately three times higher than that of GS441. According to the stability experiments’ results in the artificial gastric juice, it could be inferred (as * shown in Figure 6) that the plasma concentration curve of GS441 intersected with that of the converted GS441 from NGP-1 at the beginning of the absorption phase. The drug concentration was comparatively lower owing to the limited amount of conversion. Further, the unconverted NGP-1 was quickly absorbed in its prototype form, and GS441 was generated as an active metabolite in the blood, resulting in a higher peak concentration. The T_max_ of the converted GS441 decreased because the majority of NGP-1 was absorbed in its prodrug form, which presented favorable lipophilicity and facilitated absorption in the gastrointestinal tract.

The absorbed NGP-1 underwent rapid conversion to GS441 in vivo following absorption. The mean residence time (MRT) of the converted GS441 from NGP-1 was 6.15 h, which was approximately 2.5 times longer than the MRT of GS441 alone. This was due to the rapid absorption of NGP-1 and its swift conversion to GS441, which continually supplemented the eliminated GS441. Hence, the drug concentration was maintained for an extended duration and the MRT increased when NGP-1 was administered orally. Furthermore, the AUC_0–∞_ of converted GS441 was approximately four-fold higher than that of directly administered GS441. Meanwhile, the relative bioavailability (F_rel_) of NGP-1 was approximately 410% greater than that of GS441. Generally, the highest oral bioavailability values that can be achieved clinically using ester prodrugs are between 40 and 60% [10]. In this study, we also performed an intravenous injection experiment using GS441 in rats. The absolute bioavailability (F_abs_) was determined to be 57.7%, consistent with previous studies.

## 3. Materials and Methods

### 3.1. Chemicals and Reagents

HPLC-grade, LC-MS-grade acetonitrile (ACN) and methanol (MeOH) were purchased from Dikma Co., Ltd. (Massier Lane, Foothill Ranch, CA, USA). Normal saline was obtained from Qidu Pharmaceutical Co., Ltd. (Shandong, China). Hydrogen trifluoride triethylamine, tetrahydrofuran (THF), tertiary butyl dimethyl chlorosilane (TBSCl), dimethylaminopyridine (DMAP), imidazole, triethylamine, and ammonium acetate were obtained from Macklin Biochemical Technology Co., Ltd. (Shanghai, China). Formic acid, phosphoric acid, N,N′-carbonyldiimidazole (CDI), dicyclohexylcarbodiimide (DCC), dimethyl formamide (DMF), and isobutyric acid were purchased from Aladdin Biochemical Technology Co., Ltd. (Shanghai, China). GS441524 (GS441, MW291.3) and 6,7-dimethyl-2,3-bis(2-pyridyl) quinoxaline (QX, MW312.4) were obtained from APExBIO Co., Ltd. (Houston, TX, USA) and JiSiEnBei International Business Co., Ltd. (Hong Kong, China), respectively.

### 3.2. Animals

Sprague-Dawley rats (male, 180–220 g) were obtained from the Experimental Animal Center of Jinzhou Medical University. The animals were adapted to the standard, environmentally controlled animal room (25 ± 2 °C, relative humidity 50%, 12:12 h light/dark cycle) for 7 days. Animal care was conducted following the Guidelines for Animal Experimentation of Jinzhou Medical University and the protocol was approved by the Animal Ethics Committee of the institution (approval code: 2022110501). After the experiment, the rats were moved into the carbon dioxide death room, anesthetized with sevoflurane, and euthanized with carbon dioxide.

### 3.3. Synthesis and Confirmation of NGP-1

**Figure 7:**
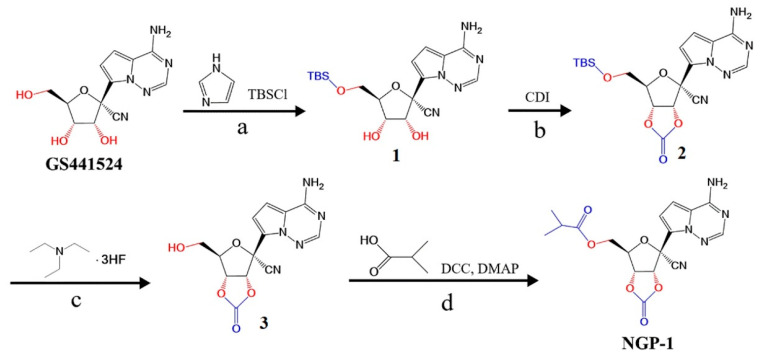
Synthetic route of NGP-1, a prodrug of GS441524, with potential anti-SARS-CoV-2 activity. Reagents and conditions: (**a**) 50 mL DMF, 0 °C, 1 h; (**b**) 100 mL ACN, 45 °C, 1 h; (**c**) 20 mL THF, 0 °C, 4 h; (**d**) 10 mL DMF, 8 h.

The general scheme of NGP-1 synthesis is shown in Figure 7 and included 4 major steps [29]. First, with the protection of TBSCl for the hydroxyl group, GS441 (34.33 mmol) and imidazole (103.0 mmol) were reacted to form compound **1**. Second, compound **1** (29.59 mmol) underwent a reaction with CDI (59.18 mmol), which was the carbonyl source, to effectuate the esterification of the ortho hydroxyl group [20]. Further, compound **2** was formed with a cyclic carbonate after undergoing separation and purification. Third, compound **2** (23.20 mmol) was made to react with triethylammonium hydrofluoride (34.80 mmol) to remove the productive base of the hydroxyl group, culminating in the synthesis of compound **3**. Finally, the hydroxyl group compound **3** (3.79 mmol) was esterified using isobutyric acid (3.79 mmol) in the presence of DCC (3.79 mmol) and a DMAP catalyst. The crude product was dissolved with ethyl acetate and washed with saturated salt water. Then, the organic layer was collected and the solvent was removed. Column chromatography was used to obtain the purified NGP-1 with DCM/MeOH (100:3, *v*/*v*) as an eluent.

The end product underwent characterization using FTIR and ^1^H-NMR techniques. Following proportional mixing and tablet formulation with dried potassium bromide, the spectra of GS441 and NGP-1 were recorded using an FTIR spectrophotometer (IRAffinity-1, Shimadzu, Kyoto, Japan) across a range of 400–4000 cm^−1^, with 20 scans per spectrum. Furthermore, GS441 and NGP-1 were dissolved in DMSO-d_6_ and analyzed using a JNM-ECA 500 MHz NMR spectrometer (JEOL, Tokyo, Japan). The ^1^H-NMR spectra were acquired with 64 scans and a relaxation delay of 60 s.

### 3.4. Purity Analysis of NGP-1

The purity of NGP-1 was confirmed through the HPLC chromatogram. After the synthesized compound was dissolved in ACN and filtered with a microporous membrane, the resulting sample was subjected to an HPLC-DAD device (ELITE LaChrom, Hitachi, Tokyo, Japan) equipped with a C_18_ column (Dikma, Beijing, China), L-2455 DAD detector, and L-2130 pump. The other chromatographic conditions are listed in Table 2. After generating the chromatogram, the areas of the peaks were integrated, and the purity was determined using the area normalization method.

### 3.5. Stability of NGP-1 in Artificial Digestive Juices

Esterification is the predominant reaction used for the generation of prodrugs, owing to the facile hydrolysis of esters by various and ubiquitous esterases present in the various matrices [18,22]. However, the presence of esters, particularly cyclic lactones, may result in instability in acidic, basic, or esterase environments. In this study, HPLC-DAD was employed to assess the stability of NGP-1 in different artificial digestive juices. The test samples were collected at various time intervals subsequent to dissolving NGP-1 in artificial gastric juice (diluted HCl with pepsin, pH = 1.5) and artificial intestinal juice (PBS with trypsin, pH = 6.8).

The HPLC-DAD system, as described in Section 3.4, was also used to simultaneously determine GS441 and NGP-1. Mobile phase A consisted of a phosphoric acid solution, while mobile phase B consisted of ACN. Gradient elution was employed to separate the components under the following conditions: 0–4 min, 30–50% B; 4–10 min, 50% B. The specific parameters for liquid chromatography are presented in Table 2. The resulting chromatogram displayed peaks corresponding to GS441 and NGP-1 after various stability samples were injected, separated, and detected. Then, the content of the objective analytes in each sample was calculated using the external standard method.

### 3.6. Pharmacokinetic Study of NGP-1 in Rats

#### 3.6.1. Pharmacokinetic Process

A pharmacokinetic study was carried out in vivo using Sprague-Dawley rats to assess the behavior of NGP-1 when administered orally through the gastrointestinal route. The rats were randomly categorized into two groups (n = 6) and subjected to a 12 h overnight fast prior to oral administration. Each group of rats received a dose of 29.1 mg/kg of GS441 or 38.7 mg/kg of NGP-1 via gavage (100 µM/kg, dissolved in 1 mL saline per 10 mg compound). After administration, 0.5 mL of orbital blood was collected at specific time intervals in an EP tube with heparin moisturizing.

All the blood samples were promptly centrifuged at 4 °C to separate the upper plasma [30]. A volume of 50 µL of plasma was subsequently transferred to a tube pre-filled with 450 µL of protein precipitant (H_2_O:ACN:MeOH = 1:4:4 containing 20 ng/mL of QX). In this study, QX was employed as an IS in detecting GS441 using LC-MS/MS. After thorough mixing, the samples underwent high-speed centrifugation. The supernatants were extracted and centrifugated again. The resulting supernatants were collected and transferred into a vial for subsequent analysis.

#### 3.6.2. LC-MS/MS Determination

LC-MS/MS was employed to detect the content of GS441 in various samples with an internal standard methodology, owing to the exceptional separation capability of LC and the high detection sensitivity of MS. The AB SCIEX QTRAP^®^4500 instrument, which comprised an LC system (ExionLC^TM^, SCIEX, Framingham, MA, USA) and a triple-quadrupole mass spectrometer with an ESI interface, was used for the experiment. To separate GS441, QX, and other components, a Kinetex^®^ C_18_ column (Phenomenex, Torrance, CA, USA) was employed along with gradient elution using water–0.05% formic acid (A) and ACN–0.05% formic acid (B). The gradient elution program was as follows: 0–1 min, 2% B; 1–2 min, 2% to 90% B; 2–3 min, 90% B; 3–3.1 min, 90% to 2% B; 3.1–5 min, 2% B. Mass spectrometric detection was performed in MRM mode and accomplished using ESI in positive ion mode with a source temperature of 550 °C, ionspray voltage of 5500 V, and nebulizer pressure of 55 psi for gas 1 and 60 psi for gas 2. The curtain gas pressure was set at 35. Additional chromatographic and the specific mass conditions are detailed in Table 2 and Table 3, respectively. The data were acquired and analyzed using Analyst 1.7.3 coupled with MultiQuant 3.0.3 (SCIEX). 

#### 3.6.3. Pharmacokinetic Fitting

According to the various concentrations of GS441 in different samples, a non-compartmental model was employed using the Drug and Statistics (DAS) version 2.1.1 software (Committee of Mathematic Pharmacology of the Chinese Society of Pharmacology, Shanghai, China) [31] to fit the plasma concentration–time curve and determine the pharmacokinetic parameters, such as C_max_, T_max_, AUC_0–t_, and t_1/2_.

### 3.7. Statistical Analysis

All data were presented as mean ± standard deviation (SD). Differences among experimental groups were analyzed by one-way analysis of variance (ANOVA) with a post hoc test of significance between individual groups, and each group was evaluated with Student’s *t*-tests. A *p* value of < 0.05 was considered statistically significant.

## 4. Conclusions

The COVID-19 pandemic outbreak has led to a significant global public health emergency. GS441, a nucleotide analog, has demonstrated marked inhibitory activity toward SARS-CoV-2. However, its poor ability to cross the cell membranes of absorption sites remains a challenge. In fact, most existing GS441 derivatives are difficult to administer orally due to their lower absorption ability and oral bioavailability. In our study, a novel prodrug of GS441 (NGP-1) was designed and synthesized with side chains of isobutyl esters and a cyclic carbonate structure, to increase the drug’s lipophilicity and oral bioavailability. The purity and stability of NGP-1 were evaluated with HPLC-DAD. Then, a precise and rapid LC-MS/MS method was developed to determine the plasma GS441 concentrations. The plasma concentration–time curve of GS441 was fitted, and a series of pharmacokinetic parameters were determined. The oral bioavailability of NGP-1 was calculated to be four times higher than that of GS441. Our findings indicate that NGP-1, a potent prodrug of GS441, has potential activity to treat SARS-CoV-2. Therefore, this compound can be conveniently administered orally, rapidly absorbed, and increasingly used by the body. This study provides important information regarding the pharmacokinetics and clinical efficacy of anti-SARS-CoV-2 medications.

## Figures and Tables

**Table 1:** Pharmacokinetic parameters of converted GS441 from NGP-1 and GS441 via gavage administration in rats (n = 6, mean ± SD).

Parameters	GS441	Converted GS441 from NGP-1
T_1/2_/h	1.53 ± 0.32	2.65 ± 0.23
T_max_/h	1.0	0.5
k_a_/h^−1^	3.57 ± 1.36	21.24 ± 3.52
C_max_/ng·mL^−1^	831.6 ± 308.4	2470.3 ± 308.8
AUC_0–t_/h·ng·mL^−1^	2316.7 ± 429.1	8431.5 ± 734.9
AUC_0–∞_/h·ng·mL^−1^	2389.2 ± 860.3	9716.3 ± 1223.7
MRT/h	2.48 ± 0.76	6.15 ± 1.78

**Table 2:** Liquid chromatography conditions for determination of purity, stability, and pharmacokinetics.

	Purity	Stability	Pharmacokinetics
Column parameters	250 mm × 4.6 mm, 5 µm	250 mm × 4.6 mm, 5 µm	100 mm × 4.6 mm, 2.6 µm
Chromatography equipment	HPLC-DAD	HPLC-DAD	LC-MS/MS
Detected wavelength/nm	240	240	- *
Column temperature/°C	30	30	40
Injection volume/µL	10	10	5
Aqueous phase (A)	water	water (with 0.1% of H_3_PO_4_)	water (with 0.05% of formic acid)
Organic phase (B)	ACN	ACN	ACN (with 0.05% of formic acid)
Elution mode	isocratic	gradient	gradient
Flow rate/mL·min^−1^	1	1	0.6
Detection time/min	10	10	5
Monitoring peak	NGP-1	NGP-1 and GS441	GS441 and QX (IS)
Quantitative method	area normalization	external standard method	internal standard method

* The detector of LC-MS/MS was the mass spectrum.

**Table 3:** Analyte-specific mass spectrometry parameters for the determination of GS441 and QX (IS).

	GS441	QX (IS)
Parent Ion/m/z	292.0	313.1
Product Ion/m/z	202.0	246.1
Declustering Potential/V	70	90
Entrance Potential/V	10	10
Collision Energy/V	20	46
Collision Cell Exit Potential/V	6	6

## Data Availability

The authors confirm that the data supporting the findings of this study are available within the article and its Supplementary Materials.

## Supplementary Materials

The following supporting information can be downloaded at: https://www.mdpi.com/article/10.3390/molecules28155634/s1, Method validation for quantitation. Figure S1: MRM chromatogram of QC sample (including 200 ng/mL of GS441 and 20 ng/mL of QX). Figure S2: MRM chromatogram of subsequent blank solvent after HQC sample injected (including 600 ng/mL of GS441 and 20 ng/mL of QX). Figure S3: Calibration curve of GS441 (1–800 ng/mL). Figure S4: MRM chromatogram of LLOQ sample (including 1 ng/mL of GS441). Table S1: Accuracy and precision of LC-MS/MS method for GS441 determination (n = 6). Table S2: Stability of GS441 under several storage conditions (n = 6). Table S3: The mean exaction recovery and matrix effects of GS441 and QX (n = 6).
