# Supplementary material for: Assessment of Purity, Stability, and Pharmacokinetics of NGP-1, a Novel Prodrug of GS441254 with Potential Anti-SARS-CoV-2 Activity, Using Liquid Chromatography
Source: Molecules. 2023 Jul 25;28(15):5634. doi: 10.3390/molecules28155634 (PMC10420250; doi:10.3390/molecules28155634)
Supplement: Supplementary file 1 [file molecules-28-05634-s001.zip › molecules-2472229-supplementary.pdf]

Supplementary Material

Method validation for quantitation.

1. Specificity and selectivity

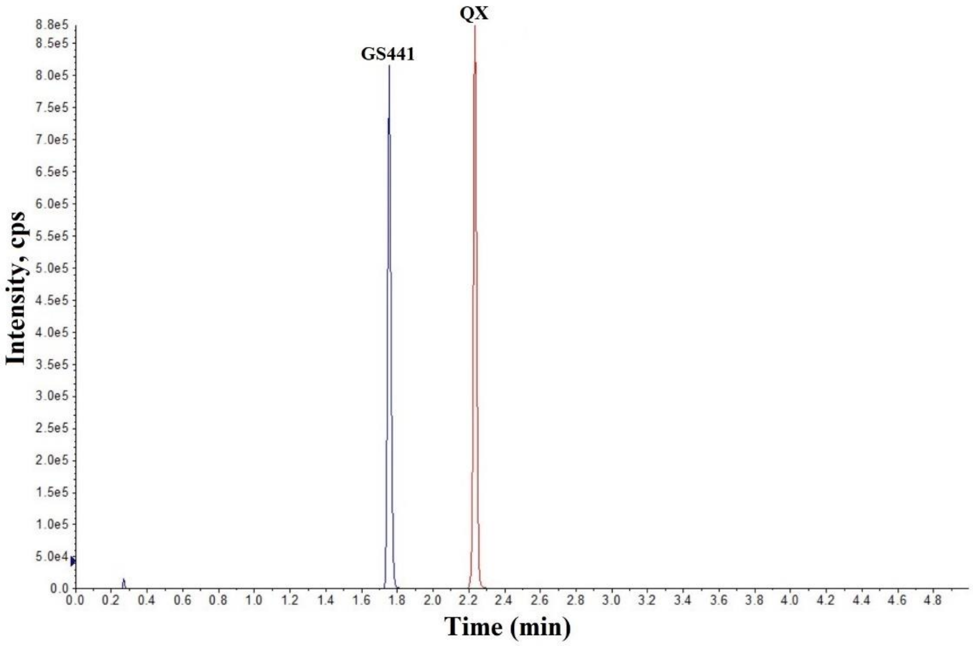

Figure S1. MRM chromatogram of QC sample (including 200 ng/mL of GS441 and 20 ng/mL of QX).

2. Carry over

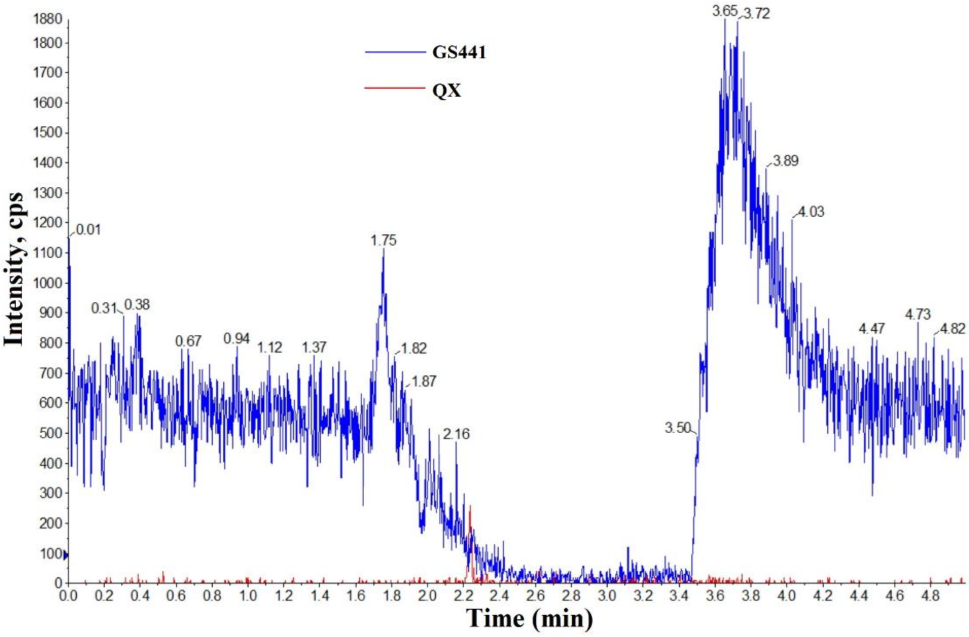

Figure S2. MRM Chromatogram of subsequent blank solvent after HQC sample injected (including 600 ng/mL of GS441 and 20 ng/mL of QX).

3. Calibration curve

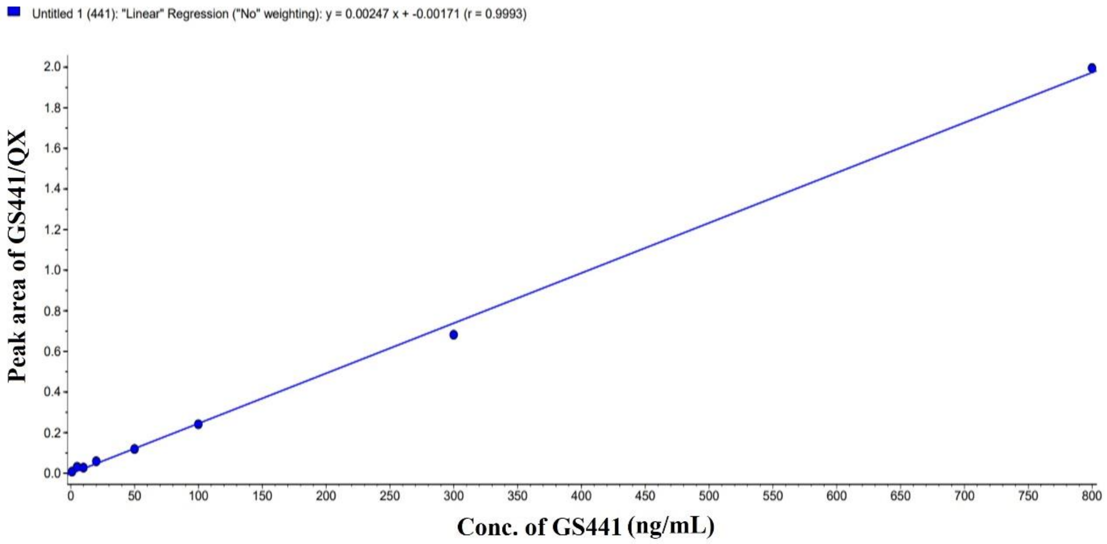

Figure S3. Calibration curve of GS441 (1-800 ng/mL).

4. LLOQ

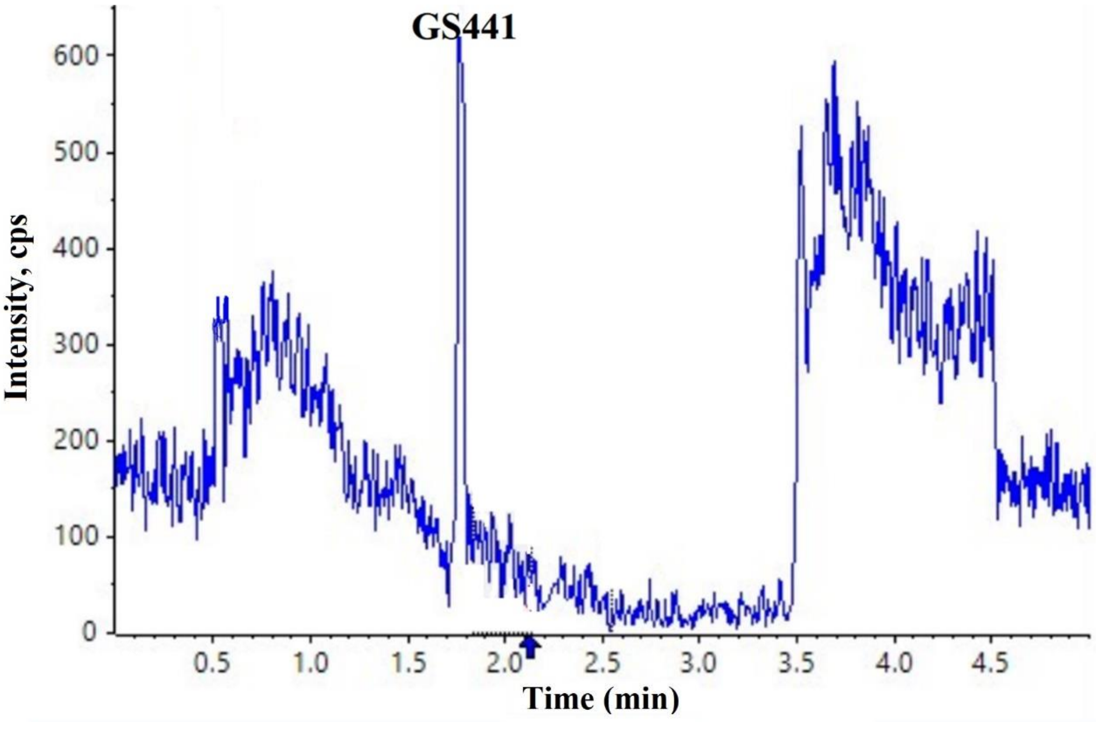

Figure S4. MRM chromatogram of LLOQ sample (including 1 ng/mL of GS441).

5. Accuracy and precision

Table S1. Accuracy and precision of LC-MS/MS method for GS441 determination (n=6).

Concentration (ng/mL)	Intra-day assay		Inter-day assay	
	Accuracy, %	Precision (CV, %)	Accuracy, %	Precision (CV, %)
2 ng/mL	93.5	6.79	110.7	8.93
5 ng/mL	105.0	4.11	103.2	7.63
400 ng/mL	102.8	1.06	97.6	4.92
600 ng/mL	98.5	1.21	93.9	5.18

6. Sample stability

Table S2. Stability of GS441 under several storage conditions (n=6).

Concentration (ng/mL)	RT 12h (CV, %)	Placed in the automatic sampler for 12 h (CV, %)	Freeze-thaw after 3 Cycles (CV, %)	Stored at -20℃ for 2 weeks (CV, %)
2 ng/mL	7.9	5.7	10.7	11.3
400 ng/mL	3.1	6.2	9.4	8.4
600 ng/mL	2.8	4.9	8.6	4.6

7. Extraction recovery and matrix effect

Table S3. The mean exaction recovery and matrix effects of GS441 and QX (n=6).

Concentration (ng/mL)	Recovery (%, mean ± RSD)	Matrix factor (%, mean ± RSD)	
		GS441	QX (IS)
2	95.23 ± 7.28	93.1 ± 1.8	101.3 ± 0.8
400	89.72 ± 11.43	100.5 ± 0.7	98.6 ± 1.7
600	90.96 ± 9.47	97.4 ± 1.1	98.4 ± 1.2
